# Reconciling the biomedical data commons and the GDPR: three lessons from the EUCAN ELSI collaboratory

**DOI:** 10.1038/s41431-023-01403-y

**Published:** 2023-06-15

**Authors:** Alexander Bernier, Fruzsina Molnár-Gábor, Bartha M. Knoppers, Pascal Borry, Priscilla M. D. G. Cesar, Thijs Devriendt, Melanie Goisauf, Madeleine Murtagh, Pilar Nicolás Jiménez, Mikel Recuero, Emmanuelle Rial-Sebbag, Mahsa Shabani, Rebecca C. Wilson, Davide Zaccagnini, Lauren Maxwell

**Affiliations:** 1 EUCANCan: European-Canadian Cancer Network, Barcelona, Spain; 2 euCanSHare: An EU-Canada Joint Infrastructure for Next-Generation Multi-Heart Research, Barcelona, Spain; 3 Centre of Genomics and Policy, McGill University Faculty of Medicine and Health Sciences, Montréal, QC Canada; 4 Heidelberg Academy of Sciences and Humanities, Heidelberg University, Heidelberg, Germany; 5 Centre for Biomedical Ethics and Law, Department of Public Health and Primary Care, Faculty of Medicine, KU Leuven, Leuven, Belgium; 6 Institute on Ethics & Policy for Innovation (IEPI), McMaster University, Hamilton, ON Canada; 7 RECODID: Reconciliation of Cohort Data in Infectious Diseases, Heidelberg, Germany; 8 ELSI Services & Research, BBMRI-ERIC, Graz, Austria; 9 CINECA: Common Infrastructure for International Cohorts in Europe, Canada, and Africa, Heidelberg, Germany; 10 EUCAN-Connect: Federated, FAIR Platform Enabling Large-Scale Analysis of High-Value Cohort Data Connecting Europe and Canada in Personalized Health, Groningen, the Netherlands; 11 School of Social and Political Studies, University of Glasgow, Glasgow, Scotland UK; 12 EuCanImage: A European Cancer Image Platform Linked to Biological and Health Data for Next Generation Artificial Intelligence and Precision Medicine in Oncology, Barcelona, Spain; 13 Social and Legal Sciences Applied to the New Technosciences Research Group, Faculty of Law, University of the Basque Country, Bilbao, Spain; 14 CERPOP, Inserm, Toulouse Paul Sabatier University, Toulouse, France; 15 Metamedica, Faculty of Law and Criminology, Ghent University, Ghent, Belgium; 16 Institute of Population Health, University of Liverpool, Liverpool, UK; 17 Lynkeus S.R.L, Roma, Italy; 18 Heidelberg Institute for Global Health, Heidelberg University, Im Neuenheimer Feld 130/3, 69120 Heidelberg, Germany

**Keywords:** Social sciences, Ethics

## Abstract

The coming-into-force of the EU General Data Protection Regulation (GDPR) is a watershed moment in the legal recognition of enforceable rights to informational self-determination. The rapid evolution of legal requirements applicable to data use, however, has the potential to outstrip the capabilities of networks of biomedical data users to respond to the shifting norms. It can also delegitimate established institutional bodies that are responsible for assessing and authorising the downstream use of data, including research ethics committees and institutional data custodians. These burdens are especially pronounced for clinical and research networks that are of transnational scale, because the legal compliance burden for outbound international data transfers from the EEA is especially high. Legislatures, courts, and regulators in the EU should therefore implement the following three legal changes. First, the responsibilities of particular actors in a data sharing network should be delimited through the contractual allocation of responsibilities between collaborators. Second, the use of data through secure data processing environments should not trigger the international transfer provisions of the GDPR. Third, the use of federated data analysis methodologies that do not provide analysis nodes or downstream users access to identifiable personal data as part of the outputs of those analyses should not be considered circumstances of joint controllership, nor lead to the users of non-identifiable data to be considered controllers or processors. These small clarifications of, or modifications to, the GDPR would facilitate the exchange of biomedical data amongst clinicians and researchers.

## Introduction

The widespread adoption of national data protection laws, including the European Union’s flagship General Data Protection Regulation (GDPR), has created debate regarding the ambit of permissible international transfers of personal data, and the appropriate legal and technical mechanisms to safeguard such transfers. These difficulties also arise relative to prior legislation. However, the high costs of non-compliance with the GDPR, and the requirement to ensure compliance with multiple competing national data protection laws have heightened pre-existing challenges [1–4]. The court-initiated adoption of new procedural requirements as preconditions to outbound transfers from the European Economic Area (EEA) to third countries outside the EEA has also exacerbated difficulties in performing outbound transfers of data from the EEA. These are further discussed in the following sections.

## GDPR

The enactment of the GDPR prompted the publication of reports from organisations representing researchers, stating that data protection legislation has created legal incertitude and high administrative costs that inhibit or outright preclude data exchange activities that are foundational to biomedical research [5–9]. Barriers to inter-institutional cooperation, for partners both within and outside the EEA, arise from the potential for the individuated participating institutions in large networks of collaborators to bear legal liability arising from the acts of the other implicated partner institutions.

## Schrems I and Schrems II

Other major data protection controversies that have prompted vigorous debate include the Court of Justice of the European Union (CJEU) determinations in *Schrems I* and *Schrems II*. These court decisions held that the Safe Harbour Decision and its successor, the EU-United States (US) Privacy Shield, were invalid. These agreements, negotiated between the EC and US regulators, enabled data transfers from the EEA to the US without requiring parties in the EEA to perform additional efforts to ensure legal compliance. The court holding in each case determined that the concerned EU-US agreement breached constitutional fundamental rights guarantees to privacy, data protection, due process, and effective remedies to which EU citizens are entitled [10, 11]. In *Schrems II*, the CJEU further held that parties in the EEA transferring data to most non-EEA countries must perform an assessment of the local legislation to determine whether the law and practice in the destination countries protected the aforementioned fundamental rights [11]. Transfers to those countries could not proceed unless appropriate safeguards were implemented to guarantee respect for such rights. This places a high administrative and legal compliance burden on health sector entities in the EEA that perform outbound international data transfers [11–14].

The European and Canadian (EUCAN) Ethical, Legal, and Social Issues (ELSI) Collaboratory is an informal collaboration established to foster discussion among the ethical and legal working groups of six distinct Horizon 2020 projects that included scientific partners in both Canada and the European Union (EU; detailed in Table 1). In this article, the EUCAN ELSI Collaboratory advances three proposals for legal reforms to facilitate the reuse of biomedical data and health data, whilst ensuring that GDPR data protection guarantees are upheld. The three models studied are a contractual approach, a data visitation approach, and a federated data analysis approach.

**Table 1:** Overview of EUCAN ELSI Collaboratory Consortia.

Short name	Full name	Regions	Datatypes	Disease or system focus	Approach to data reuse beyond the consortium	Mission	Years
CINECA	Common Infrastructure for National Cohorts in Europe, Canada, and Africa	Africa, Europe, North America	Genetic, phenotypic, and biomolecular data	Any	Federated analysis; Create synthetic data for download	Develop a federated, cloud-based infrastructure for making genomic and biomolecular data available	2019–23
EUCANCan	European-Canadian Cancer Network	Europe, North America	Genomic and phenotypic data	Cancer genomics	Federated cloud-based clinical data infrastructure	Federated Network of Aligned and Interoperable Infrastructures for the Homogeneous Analysis, Management, and Sharing of Genomic Oncology Data for Personalized Medicine	2019–22
EUCAN-Connect	Federated, FAIR platform enabling large-scale analysis of high-value cohort data connecting Europe and Canada in personalized health	Europe, North America	Data from cohort studies	Cardio-metabolic, musculoskeletal, respiratory health and disease	Federated analysis	Federated analysis	2019–23
EuCanImage	A European Cancer Image Platform Linked to Biological and Health Data for Next Generation Artificial Intelligence and Precision Medicine in Oncology	Europe	Imaging data (cancer imaging) and non-imaging data (associated clinical and other health data)	Cancer	Hybrid (centralized and federated)	Enable the reuse of high-dimensional cancer imaging data and associated non-imaging data to enhance the potential of AI in oncology	2022–24
euCanSHare	An EU-Canada Joint Infrastructure for Next Generation Multi-Study Heart Research	Europe, North America	Imaging, biomarker	Cardiac imaging	Centralized, cloud-based infrastructure with the ability to download data or use data in the cloud	Support biomarker validation, cardiovascular risk assessment, knowledge discovery, public health	2018–22
ReCoDID	Reconciliation of Cohort Data in Infectious Diseases	Europe and the Americas	Health research data	Emerging infectious diseases (arboviruses, specifically, Zika, Chikungunya, and Dengue) and COVID-19	Centralized, cloud-based infrastructure with the ability to download data or use data in the cloud	Create cloud-based infrastructure and governance for the reuse and potentially link clinical of epidemiological and high-dimensional laboratory data	2019–23

Part 1. Joint controllership contracts

EEA and international data sharing efforts often use contracts to define the boundaries of permissible data sharing and data use between project partners and others authorised to use data (e.g., third-party service providers, downstream researchers). These contracts ascribe responsibilities for providing select services to respective partners in the collaboration, and apportion the consequences and costs of non-compliance.

The design of these contracts is case-specific. Specific parties take responsibility for delivering services that fall within their circle of competence and that are associated with their role as defined in the contract. These contracts usually shield select parties from certain forms of liability. Expert advisors or operational staff might be shielded from liability in their personal capacities to encourage their participation in creating and operating international data sharing infrastructure, especially where their participation is performed on a volunteer basis. Entities that operate data hosting platforms are often shielded from liability if users of their platform misuse the platform in a manner that causes harm. Consortium partners who are responsible for developing and implementing a limited portion of a larger infrastructure often use contracts to limit their liability to those elements that fall to their direct control.

The preceding practices might superficially appear to limit the recourse available to data subjects. In practice, however, enabling distinct parties to align the breadth of their potential liability to that of their specified responsibilities incentivises more partners to lend their expertise to data-sharing efforts or to the design of a platform. If collaborating research institutions, individual expert contributors, citizen volunteers, or third-party service providers stand to bear all liability for the data protection non-compliance of a data sharing or platform design effort, regardless of their contribution to such non-compliance, the legal risks will curtail their participation in such efforts. Conversely, enabling parties in such an exchange to apportion respective responsibilities through a legally binding contract, and limiting the liability of such parties to the ambit of their contractual commitments, encourages a larger number of stakeholders to contribute [15–18].

The General Data Protection Regulation (GDPR) uses the concepts of ‘controller’ and ‘processor’ to determine the respective legal obligations of distinct actors engaged in the processing of personal data. Controllers determine how personal data will be processed. These actors must ensure compliance with the substantive and procedural requirements of the GDPR. Processors are required to implement the instructions of controllers, but are not held responsible for most aspects of GDPR compliance. Therefore, the law directs most legal compliance obligations to the ‘controllers’ that choose how information is to be used, and only hold the ‘processors,’ their delegates in implementing such choices, to listen to instruction and to discharge select other minor duties [19].

The law can also hold collaborators that together determine the use of data to be ‘joint controllers.’ This is a special legal status that arises if multiple legal entities or individuals together share the distinct duties, responsibilities, and behaviors that are incumbent on controllers. Joint controllership would arise if multiple actors together performed common processing activities directed to the same data for shared purposes. This status would not arise if multiple actors transferred data to one another to process for independent purposes.

In sum, therefore, delineating which actors are considered to be controllers, and which are considered to be joint controllers, is central to performing sound GDPR compliance. Difficulties in determining which actors are controllers, or joint controllers, can lead such actors to neglect their responsibilities, or to be held non-compliant for obligations that such actors did not consider themselves bear.

The GDPR builds on the data protection rules enshrined in its precursor statute, the DPD, often mirroring its edicts. One of its notable departures from the DPD is to introduce a definition of joint controllership and to define the liability rules applicable to joint controllers [19, 20].

The text of the DPD did not stipulate whether it was possible for multiple entities to be considered ‘joint controllers’ and therefore, to share in the responsibilities for data protection compliance as a collective group [20]. Courts later interpreted the law to allow for joint controllership. Joint controllership in the DPD was interpreted broadly, recognizing a large potential range of legal entities and individuals, including social media platforms and platform users, as joint controllers. However, the courts held that the responsibilities of each joint controller – and their prospective legal liability – were asymmetric in scope. Such prospective liability was limited to activities that the concerned actor performed or for which such actor otherwise bore the burden [21–23].

In contrast, the GDPR defines joint controllership, and requires joint controllers to adopt arrangements that apportion their respective responsibilities for each part of the overall data controllership effort [19]. The GDPR further stipulates that each joint controller is subject to joint and several liability for the overall data processing effort [19]. Despite the clear delineation of responsibilities between joint controllers using contracts, each joint controller remains liable for all elements of data protection non-compliance arising from other joint controllers’ acts. A joint controller can only unburden itself of such liability by demonstrating “that it is not in any way responsible for the event giving rise to the damage” [19]. The assessment of whether an actor is a joint controller – and can be held liable for the data protection non-compliance of other actors – is left to the determination of supervisory authorities and courts [19].

The DPD performed a nuanced balancing of the respective roles of different collaborators in data processing activities. In a sense, the DPD implied contractual relationships that parties might have negotiated into their respective data protection obligations. In contrast, the GDPR disincentivizes data sharing and platform creation efforts from investing in private law arrangements that apportion relative responsibilities between collaborators. Regardless of the arrangement, each collaborator bears full legal responsibility for all data processing activities that the collective of joint controllers performs [19].

Member projects of the EUCAN ELSI Collaboratory have had considerable success in creating platforms and networks to share data. These efforts were initiated according to the DPD, prior to the enactment of the GDPR and the associated change in joint controllership rules. The transition from the DPD to the application of joint and several liability under the GDPR has slowed or prevented our efforts to enable international data sharing to facilitate the reuse of the data in the health sector.

To achieve heightened success in stimulating inter-institutional and international collaboration that engenders an improved standard of data protection, the following interpretation of the GDPR can be recommended. The law should empower prospective joint controllers to apportion their respective responsibilities according to contractual terms of their own determination. It should not hold such joint controllers to a standard of joint and several liability. If joint controllers do not enter into contracts that define their respective data protection responsibilities, supervisory authorities and courts should interpret their respective responsibilities in concordance with the role of each in the overall arrangement, as arises from the factual situation. This approach reaffirms the rule in the DPD [20]. It could be incorporated to the GDPR through an amendment, through jurisprudence confirming its continued application, or through EDPB guidance. This position could also be confirmed in a GDPR Code of Conduct directed to healthcare or research data processing [14].

Further justification for this proposed reversion is the following. The GDPR made the explicit choice to alter the DPD’s liability rule, so as to subject joint controllers to joint and several liability. Recital 146 of the GDPR explains this choice, stipulating that: “Data subjects should receive full and effective compensation for the damage they have suffered. Where controllers or processors are involved in the same processing, each controller or processor should be held liable for the entire damage.” [19]

This liability rule dissuades organisations from collaborating in performing data processing, and providing ancillary services. Indeed, numerous organisations will choose not to participate in a collective effort if it requires them to bear the compliance risk associated to the non-compliance of the other collaborators. However, inter-organisational collaboration is crucial both to performing data sharing, and to guaranteeing an appropriate degree of data protection in performing such sharing. The punitive liability rule articulated in the GDPR might indeed provide tortfeasors with a wider range of defendants to pursue. But it incentivizes organisations to opt out from large-scale efforts to securely share data for fear of liability, reducing collaboration in sharing data and in ensuring that such sharing achieves an appropriate standard of data protection.

This conclusion reflects the experience of the EUCAN ELSI Collaboratory. It is further supported by a broad literature that demonstrates the poor public policy outcomes of joint and several liability rules in numerous contexts. The demonstrated effects of adopting such rules include taxing the purse of large public-sector bodies that contribute in a small manner to the non-compliance of smaller actors, exposing these large public defendants to unsustainable judgment costs, and disincentivizing risk-averse but highly specialised actors, including consultants, SMEs, or academic collaborators, from contributing their expertise to collaborative endeavors, for fear of bearing liability for the acts of others [15–17]. Joint and several liability rules disfavor inter-organisational collaborations relative to established monopolists that can internalize the entire data processing value-chain into the boundaries of a single firm. Ironically, monopolies in data processing count among the societal harms that the GDPR was implemented to counteract [19].

Part 2. Secure Data Processing Environments for International Collaboration

The international data transfer rules of the GDPR pose difficulties for international collaborations in health and biomedical research. The structure of these rules, as further detailed below, can outright preclude some organisations from performing, or receiving, international transfers of personal data from the EU/EEA, either because it is not possible for the transfer recipient to meet the requirements of EU law, or because it is too burdensome to perform the required compliance activities. According to the internal logic of data protection law, such transfer requirements are implemented to ensure that the fundamental rights guarantees accorded to EU citizens are not compromised if data is transferred outside of the reach of EU law. Responses to this public policy choice are divided. Some contend that the onus is on third jurisdictions to adopt data protection standards that are aligned to those of the EU, and that data flows from the EU/EEA should rightly be impeded until such alignment is ensured [24]. Others hold that precluding data flows from the EU/EEA to third countries denies those in Europe the benefits of international health research, and that political matters should not arrest such crucial information flows [1, 14]. No resolution to this schism is forthcoming. Our proposal, detailed below, advocates in favor of a ‘third way’: the creation of secure processing environments that enable researchers from third countries to access data according to organisational and technological safeguards that ensure that the fundamental rights recognised in the EU are applied to such processing activities [14, 25].

Instead of having the effects of the law follow the data as it moves to third parties outside the EU/EEA, according to the GDPR transfer rules, this approach would require third parties outside the EU/EEA to restrict their data use to processing environments that incorporate EU fundamental rights guarantees to their intrinsic structure.

To this end, the European Commission (EC) should elaborate the legal treatment of cross-border ‘data visitation’ arrangements. Data visitation is the provision of access to data through a controlled, secure processing environment that enables data analyses in the cloud, without enabling data download [26]. In this model, the EC, or delegates thereof, would determine the technical and organisational specifications of secure processing environments for international collaboration in health data analysis. These decisions would establish the contractual and administrative prerequisites for obtaining access to a secured ‘data visitation’ space. Such decisions would also confirm the appropriate technical approach to maintaining platform security, the methods used to ensure the appropriate use of data accessed through secure processing environments, and the rules that must be followed prior to uploading data to a secure processing environment. In contrast to data transfers enabled through the purely contractual approach, the use of a secure processing environment enables actors from jurisdictions that cannot receive transfers of personal data from the EU or the EEA to collaborate in international research [12].

## Adequacy decisions for data transfers outside of EEA

Currently, certain jurisdictions cannot receive transfers of personal data from the EU or the EEA because of the legal determinations made in *Schrems I* and *Schrems II* [10–12]. One principal holding of these cases is that data exporters in the EU or the EEA that transfer data to jurisdictions that the EC has not deemed to be ‘adequate’ must perform an assessment of the destination country’s legislation and practice. This assessment verifies whether the recipient jurisdiction can ensure compliance with the fundamental rights to privacy, data protection, due process, and effective remedies to which EU citizens are entitled as a matter of constitutional law [11, 12]. This burden is disproportionate relative to the limited legal compliance resources available to data exporters [2, 11, 12].

If the assessment demonstrates that the law and practice of the recipient jurisdiction provides a standard of data protection that is on par with that of the GDPR, data can be transferred to that jurisdiction in reliance on one of the additional transfer mechanisms established in the GDPR [11, 12].

If the legal assessment demonstrates that the local law and practice of the recipient jurisdiction cannot achieve a standard of data protection compatible with the foregoing fundamental rights, the following consequences ensue. Transfers from the EU or the EEA to the concerned jurisdiction can still be performed if supplementary measures are adopted to remedy the deficiencies in the rights guaranteed to data subjects [11, 12]. These include “contractual, technical, or organisational” measures [12]. That said, the supplementary measures are generally limited to technical approaches. In most jurisdictions in which law and State practice do not align with the aforementioned fundamental rights of EU citizens, the implementation of contracts or organisational practices are unlikely to succeed in bolstering the protection of EU citizens from incursions on their fundamental rights. In such instances, additional technological safeguards can be implemented to prevent acts that breach the fundamental rights of EU citizens [12].

Sometimes, none of the foregoing approaches are suitable to enable transfers of data from the EU or the EEA to third jurisdictions. Certain institutions would not be able to conform to the requirements of the GDPR’s transfer mechanisms because of restrictions in domestic law applicable to them, regardless of whether or not the transfer could be performed in accordance with the requirements of the GDPR [27]. Further, numerous intended uses of data are incompatible with the additional technological safeguards required to transfer data to jurisdictions that could not ensure fundamental EU rights guarantees [2, 11–13].

To remediate these difficulties, international collaborators barred from receiving data transfers from the EU or the EEA could perform the analysis of such data within a secure data processing environment established using EU technical infrastructure, that itself guarantees respect for the fundamental rights of EU citizens through its platform-integrated technological and organisational safeguards [26, 28]. Organisational safeguards include access controls, audits of platform use, or data use practices that platform personnel and external users are required to respect.

We have yet to determine whether the GDPR would construe non-EU or non-EEA access to data performed through a web browser or other portal as an international data transfer. Limited jurisprudence has assessed such questions in the past, however, there is no consensus position on this legal issue at present (Lindqvist).^1^ The EDPB, for its part, considers access to data in the EU from third countries to constitute an international transfer of data.

Providing data access to users in other jurisdictions through a secure data processing environment should not be construed an international transfer of personal data. The EC could confirm that the processing of personal data on a secure data processing environment that follows approved technical specifications constitutes personal data processing, but is not subject to the additional GDPR international data transfer rules. The secure data processing environment ensures that the concerned data processing presents the same balance of risks as other data processing activities that remain in the territorial and jurisdictional boundaries of the EU and the EEA. There is consequently no need to place additional reliance on the GDPR’s transfer safeguards to mitigate additional risks that international collaboration might otherwise create, if the data were to be transferred to third countries, rather than being processed in a secure data processing environment [12, 14, 26].

This outcome incentivises data custodians to create and to utilise secure data processing environments that are equipped with state-of-the-art audit logs and other technical safeguards, rather than to disclose data to downstream users in other jurisdictions.^2^ In lightening the legal compliance burden associated with enabling data visitation, relative to data transfer, legislators and regulators create incentives for data custodians to develop platforms that hard-code local regulatory requirements into their functioning [26]. However, it must be acknowledged that some forms of data processing might not be susceptible to this solution, such as analyses that are reliant on being able to export identifiable outputs of data processing off of the platform, or those that are contingent on the upload and combination of external datasets.

To establish this solution, the EC, the EDPB, or independent experts drafting a Code of Conduct, should stipulate the technical requirements applicable to secure data processing environments. Public bodies or private bodies should be authorised to create secure data processing environments and operate them according to the technical specifications provided. Compliance with these requirements on the part of upstream data contributors, operators, or downstream data users should be assumed to ensure compliance with the GDPR. Such activities should be construed as the GDPR-regulated processing of personal data, but not as the international transfer of personal data, even where select contributing nodes, operators, or user nodes are situated outside the EU or EEA. To further stimulate innovation in the functioning of secure data processing environments, there should be a mechanism to enable the technical specifications of alternate designs for secure processing environments to be proposed, formally approved, and published in public repositories, enabling their future use. Certification could also be offered to the operators of secure processing environments, or to in-environment data users that conform to the requirements thereof, to further confirm their compliance with the applicable requirements.

Part 3. Federated data analysis

Federated data analysis has been proposed as a panacea for the challenges inherent in performing the centralised storage and analysis of large datasets, and those arising from the centralised storage and analysis of regulated datasets [29, 30]. Ambiguities remain as to the data protection compliance outcomes of federated data analysis [30, 31].

Federated data analysis leaves most of the responsibilities for data storage and data analysis under the technical and organisational control of the data’s original host institution, whilst enabling all participating institutions to share in the output results of each contributing node’s local analysis. This is often achieved through the use of distributed, rather than centralised, computing [29, 30, 32–34].

Federated data analysis resolves challenges in bringing together the significant storage and compute resources required to perform the large-scale statistical analysis of data. The individual-level data that each node contributes to a federated data analysis can be stored and analysed using the concerned node’s own technical architecture. There is no need for a singular central node to possess the considerable infrastructure required to store large quantities of individual-level data, or to perform large-scale data analysis. This burden is shifted, piecemeal, from the central node to each of the smaller nodes that comprise the network of participants in the overall analysis [30, 33].

From a compliance standpoint, federated data analysis is often lauded as a compromise position between the total non-disclosure of data, and the resource-intensive negotiation of data disclosure for regulated data [35].

In performing the federated analysis of data, cooperating institutions disclose the non-identifiable outputs of local or distributed data analysis, such as statistical relationships between variables or the local inputs into a larger effort at training a machine-learning algorithm (e.g., model weights). These are then brought together, physically or virtually, from the participating nodes. Each node benefits from the output results of the federated analysis, without disclosing its regulated data to the other nodes [29–31, 34].

From the perspective of the GDPR, debate exists as to whether or not federated data analysis methodologies entail the processing, transfer, or joint controllership of identifiable personal data. This determination is dependent on the technical details of the form of federated data analysis adopted. Nonetheless, the experience of the EUCAN ELSI Collaboratory provides select insights in this respect. The following section is intended to provide our interpretation of applicable law, and to articulate select public policy justifications that favor this interpretation. It also argues that the EC, or regulators (e.g. the EDPB or national supervisory authorities) should provide guidance that helps in the ascription of GDPR roles to the actors engaged in a network of federated data analysis, so that common approaches can be adopted on this issue throughout the European Union. This creates heightened clarity for data subjects, GDPR-regulated parties, and for the downstream users of the non-identifiable outputs of federated analysis.

Federated data analysis methods that do not entail the disclosure of identifiable personal data to other participating nodes should not be interpreted as constituting either ‘joint controllership,’ nor as involving GDPR-regulated transfers of identifiable personal data between participating nodes. Users of a federated data analysis platform enabling requesting parties to obtain the results of queries, which do not constitute identifiable personal data, should not be construed as joint controllers or data transfer recipients, either [21–23]. This is especially true if the participating nodes utilise standard-form contracts to define the privileges and responsibilities of platform users, and if technical safeguards are used to delimit and to audit downstream users’ use of the federated data analysis platform.

It is our understanding that this is the correct interpretation of existing EU law. However, ambiguities as to the circumstances in which the utilisation of a federated data discovery or data analysis platform constitutes personal data processing, the international transfer of identifiable personal data, or creates a situation of joint controllership, remain.

EU legislators or regulators, such as the EC or the EDPB, should confirm that reliance on federated data analysis methods do not constitute international data transfers, nor cause joint controllership to arise. These details could also be elaborated through a GDPR Code of Conduct. This should be the case so long as no personal data is transferred amongst participating nodes or to external users, and so long as technical controls and audit measures are implemented to delimit the autonomy of nodes and end users so as to prevent them from “[determining] the purposes and means of the processing of personal data” [19].

The policy justifications for providing such confirmation are the following. The sharing, joint analysis, and transfer of full datasets compiled from identifiable personal data is cost-intensive from the standpoint of regulatory compliance. It has the potential to create risks to the rights and to the freedoms of the affected individuals, both in their capacities as EU citizens bearing fundamental rights, and in their capacities as GDPR data subjects.

Institutions implement federated data analysis platforms for numerous different reasons. Some are for reasons relating to technological or organisational resources. That is, it is often more cost-effective to adopt federated approaches to performing data analysis and data exchange [29–31, 34]. Each of the nodes participating in the analysis must possess a measure of technical infrastructure and specialist staff, however, the burden that such analysis might otherwise place on a central coordinating node is much diminished [33].

Nonetheless, one of the major benefits of federated data analysis is to create compromises from a regulatory compliance standpoint. Institutions reduce the data protection risks inherent in their data uses, through the transfer of the non-identifiable output results of local data analyses rather than the identifiable input data. In doing so, these institutions reduce the scientific utility of the concerned data, and have a more limited potential to ensure the accuracy of their results, because it is not possible for them to access the raw data that are inputs into the analysis [29–31, 33, 34].

For public policy reasons, this should be deemed the analysis of non-identifiable, non-personal data. This incentivises institutions to implement federated data analysis methods, and therefore to benefit from more cost-effective data analysis (from a compliance standpoint) in exchange for accepting certain trade-offs, such as losing the potential to view and interrogate the underlying data that contributed to analyses. Institutions will adopt methods of data analysis which minimise the residual risks to individual privacy and to individual data protection rights.

Institutions will invest in the exchange of identifiable personal data and bear the associated costs of compliance in those circumstances in which the nature of the intended data uses requires them to use identifiable personal data. Institutions will implement federated methods of data analysis in those circumstances in which the added scientific utility of exchanging identifiable personal data does not justify bearing the added compliance costs of exchanging the full identifiable data [29–31, 33, 34].

For these reasons, it is recommended that legislatures and regulators confirm that the implementation and use of federated data analysis mechanisms does not constitute the processing of identifiable personal data on the part of end-users, so long as these end-users do not obtain access to identifiable personal data through these data processing activities. It is also recommended that legislatures and regulators confirm that end-users and local nodes do not constitute joint controllers, nor recipients of international data transfers. This remains contingent on no identifiable personal data being shared between participating nodes. It also remains contingent on individual nodes, and end-users, not “[determining] the purposes and means of the processing of personal data” [19].

To better substantiate these concepts, regulators should collaborate with technical experts to release empirical methods and metrics for confirming that no identifiable data is shared through a federated data analysis, and for demonstrating that the ‘purposes-and-means’ test does not create a situation of joint controllership [36]. This could be achieved through the adoption of EDPB guidelines, a sector-specific Code of Conduct, or through a legislative amendment to the GDPR on the part of the EC. Such guidance could outline the circumstances in which participating nodes could be construed as controllers, joint controllers, processors, or as unregulated users or recipients of data that does not constitute identifiable personal data [14]. Regulators could perform audits of compliance, require parties to undergo audits of compliance in this respect, or could provide optional verification and certification mechanisms to support efforts to demonstrate compliance in this complex area.

It is recommended that nodes that process personal data as part of a larger federated data analysis network be construed as processing identifiable personal data relative to their own personal data processing activities alone, without being construed as joint controllers of the personal data that the other participating nodes process. It is also recommended that the nodes responsible for bringing together, processing, or otherwise receiving the non-identifiable outputs of a federated data analysis not be construed as the controllers or processors of personal data. This achieves the favorable balance of incentives detailed above. It encourages participants to construct federated data analysis networks that preclude the transfer of personal data between participating nodes, obtaining the results of personal data processing without incurring risks to data protection and privacy.

## Conclusion

The GDPR has been met with fanfare amongst international scientific research communities. It provides enforceable informational rights to citizens and publics. It also articulates a vision of digital societies that creates obligations to engage in the accountable and equitable use of information, whilst also enabling open and egalitarian downstream access to such data [37].

The adoption of data protection legislation can stimulate heightened public contribution to scientific endeavor, in guaranteeing research participants a common entitlement to the responsible stewardship of their data. Yet, data protection law as now implemented fails to embody these high aspirations. The restriction of international data flows, and the intensive administrative and procedural burdens integrated to data protection legislation strain the resources of clinicians, researchers, and other institutions dedicated to the prosocial use of biomedical data. Clinicians and researchers in the Global South are deprived of cost-effective participation in international efforts to leverage existing data commons for clinical and research purposes. The application of data protection norms to all data processing activities creates potential challenges to the perceived legitimacy of existing institutions dedicated to the practical administration of rights in data, such as Research Ethics Committees (RECs) and health institutions’ data custodians.

The salience of these critiques does not detract from the GDPR’s importance as a mechanism directed to creating and operationalising both local and international standards regulating acceptable information uses. But, to achieve the ambition of incentivising, enabling, and regulating information use throughout its lifecycle, considerable future expansion on the GDPR will be required.

This requires the creation of new public-sector institutions, markets for public services, sector-specific legislation, and international covenants. More targeted programs of regulatory intervention are also requisite, such as the creation of regulatory agencies, the strategic use of subsidies and taxation schemes to create context-appropriate behavioral incentives, and the promulgation of voluntary and mandatory licensing schemes to promote transparency [38].

Pending this languorous emergence of appropriate public and private mechanisms to regulate information use in a context-appropriate manner, our three proposed reforms constitute apt mechanisms to enable the full continued application of the GDPR to regulated actors, whilst enabling the cost-effective downstream use of personal data at scale. These amendments provide regulated actors with certitude as to the legal compliance of their data processing activities, and the boundaries of their respective obligations. It is our hope that these should further the goal of rendering data “as open as possible, and as closed as necessary” [39].

## Footnotes

1 There is statutory support for the idea that not all data processing activities with an extraterritorial component can be considered international transfers of data. It is most notable that the legislature has chosen to refer to data transfers as a concept distinct from, e.g. extraterritorial data processing operations. It therefore stands to reason that not all data processing operations with an extraterritorial element need constitute data transfers according to GDPR Chapter V. The GDPR defines data processing. It does not define data transfers, thus explicitly leaving the definition of this concept to courts and to regulators.

2 Data custodians are organisations and persons that assume operational responsibilities for holding data in a secure manner and facilitating its secure provision to downstream users for authorised purposes. This characterisation is agnostic to the GDPR role of the concerned organisations and persons, though these might often be considered controllers in some circumstances, data processors in others, and in certain instances, the non-regulated agents of a controller or a processor.
